# Pediatric residents’ attitudes towards and experiences with collaboration with primary and secondary schools in the United States

**DOI:** 10.3352/jeehp.2018.15.3

**Published:** 2018-01-29

**Authors:** Jeffrey D. Shahidullah

**Affiliations:** Department of Pediatrics, Robert Wood Johnson Medical School, Rutgers University, New Brunswick, NJ, USA; Hallym University, Korea

The American Academy of Pediatrics (AAP) has clearly emphasized the importance of pediatricians communicating and collaborating with primary and secondary schools on the clinical care of their patients. The AAP has published the seventh edition of “School health: policy and practice” [1], which outlines specific competencies and roles that pediatricians should undertake in coordinating care with schools and school providers, including nurses, counselors, school psychologists, teachers, and administrators. The AAP endorses the need for collaboration with schools in other ways as well, such as through their practice parameter guidelines for specific behavioral health conditions; for example, the AAP guidelines for attention-deficit/hyperactivity disorder (ADHD) emphasize that a consideration of the school environment, program, or placement should be part of any treatment plan [2]. The purpose of this opinion piece was to assess residents’ attitudes towards and experiences with communication and collaboration with schools in a pediatric residency training program at Geisinger Medical Center in the United States.

All residents included in this study provided informed consent for participation in the study. It was approved by the Institutional Review Board of Geisinger Medical Center (IRB no., 2015-0432).

The survey participants consisted of 40 of the 42 pediatric residents at Geisinger Medical Center, Danville, Pennsylvania, USA (14 of the 14 first-year residents, 13 of the 14 second-year residents, and 13 of the 14 third-year residents). The 2 missing residents were excluded by chance (scheduled vacation or off day). Surveys were administered during residents’ morning report time slot during the July 2016 to July 2017 training year. The participants completed the Attitudes and Experiences in School Health Collaboration survey (Table 1). This survey consisted of 9 total items and took approximately 6 minutes to complete. Data were analyzed descriptively to evaluate how often the participants endorsed the 4-point Likert-type scale items pertaining to “attitudes” and the items pertaining to “experiences” in school health collaboration. Item no. 6 was an open-ended question asking participants to list the barriers that they perceived in collaborating with school personnel. (“What barriers, if any, are there in collaborating with school personnel?”) These items were grouped into a total of 6 categories that were created for each set of similar responses.

The survey results are presented in Table 1. Raw data were available from Supplement 1. More than half of the respondents collaborated and/or communicated with school personnel. Four-fifths strongly or very strongly felt that collaboration/communication with schools contributed to improved patient care. The most important topic of communication with schools was reported to be ADHD.

Based on the results from the open-ended item, the following themes, which were reflected by more than 1 response, were identified: lack of time (n= 22), incompatibility of work schedules (n= 7), do not know who to contact (n= 8), do not know how to contact (n= 6), perception of schools as unable to provide adequate support (n= 4), and privacy/Health Insurance Portability and Accountability Act concerns (n=2). Specific responses are available in Supplement 1.

The results should be interpreted in light of the limitations of this study. The survey instrument was developed by the author, and a psychometric validation of the measure was not conducted due to the small sample size. Data were self-reported. Although these findings are from a single pediatric residency training program in the United States, they offer some interesting findings and suggest the need for further exploration. Residents generally recognized the value of collaborating/communicating with schools regarding patient care and believed that doing so contributed to a higher standard of care (Table 1). Early exposure to the local schools and school personnel through school-based immersion experiences should be provided through methods such as shadowing, presentations, and shared-care projects with medical and school providers. Activities of this type may provide an imprinting function by demonstrating the relevant school personnel and through the choice of a topic that is ideal for collaboration. Sufficient time for communicating with school teachers, school nurses, counselors, or school psychologists should be allocated during the pediatric resident training program.

## Figures and Tables

**Table 1.** Attitudes and experiences in school health collaboration (N=40)

Items	No. (%)
How knowledgeable are you about school special education (individual family service plans, individualized education plans) and general education (504 plans) student support services?	
Very knowledgeable	0
Knowledgeable	13 (32.5)
Somewhat knowledgeable	21 (52.5)
Not at all knowledgeable	6 (15)
How important (to you) is it that you collaborate/communicate with school personnel (teachers, counselors, school psychologists, administrators) around service provision for a patient/student?	
Very important	17 (42.5)
Important	18 (45)
Somewhat important	5 (12.5)
Not important	0
How often do you collaborate/communicate with school personnel around service provision for a patient/student?	
Often (1–2 times per week)	0
Sometimes (1–2 times per month)	5 (12.5)
Rarely (1–2 times per year)	21 (52.5)
Never	14 (35)
If you collaborate/communicate with school personnel, what profession typically is that (all that apply)^a)^?	
Administrator	2 (7.7)
Counselor	11 (42.3)
Nurse	12 (46.2)
School psychologist	7 (26.9)
Teacher	17 (65.4)
If you collaborate/communicate with school personnel, what topic is it typically on (all that apply)^a)^?	
ADHD (attention-deficit/hyperactivity disorder)	17 (65.4)
Behavior problems	13 (50)
Learning/academic problems	12 (46.2)
Medical issues (asthma, etc.)	13 (50)
Medication issues	9 (34.6)
Other mental health problems	1 (3.9)
Other (Individualized Education Program forms)	1 (3.9)
How does this collaboration/communication typically occur (all that apply)^a)^?	
Email	7 (26.9)
Fax	8 (30.8)
In-person	2 (7.7)
Phone	16 (61.5)
Postal mail	15 (57.7)
How likely are you to collaborate/communicate with school personnel in the future?	
Very likely	16 (40)
Slightly likely	16 (40)
Slightly unlikely	7 (17.5)
Very unlikely	1 (2.5)
How strongly do you feel that collaboration/communication with schools contributes to improved patient care^b)^?	
Very strongly	15 (53.4)
Strongly	8 (28.6)
Somewhat strongly	4 (14.3)
Not very strongly	1 (3.6)

Percentages do not add to 100 due to non-responses.

a) 26 total respondents.

b) 28 total respondents.

## Supplementary material

Supplement 1. Data files are available from https://doi.org/10.7910/DVN/7FC9XY
